# Independent evaluation of the effectiveness of IsoCal in improving image center accuracy on Varian TrueBeam and Clinac machines

**DOI:** 10.1002/acm2.12402

**Published:** 2018-06-29

**Authors:** Weiliang Du, Song Gao, Wei Jiang, Rajat J. Kudchadker

**Affiliations:** ^1^ Department of Radiation Physics The University of Texas MD Anderson Cancer Center Houston TX USA; ^2^ Yantai Yuhuangding Hospital Qingdao University School of Medicine Yantai Shandong China

**Keywords:** image center, IsoCal, quality assurance, Winston‐Lutz test

## Abstract

Modern medical linear accelerators (linacs) are often equipped with image guidance systems that are capable of megavolt (MV), kilovolt (kV), planar, or volumetric imaging. On Varian TrueBeam linacs, the isocenter accuracies of the imaging systems are calibrated with a procedure named IsoCal. On Clinac series linacs from Varian, installation of IsoCal is optional and the effects of IsoCal on the imaging systems can be turned on or off after the IsoCal procedure is performed. In this study, we report on the effectiveness of IsoCal in improving the coincidence of the image centers with the radiation isocenter, using an independent Winston‐Lutz (WL) method to locate the radiation isocenter. A ball‐bearing phantom was imaged with 2D MV, 2D kV, and cone beam computed radiography systems on two TrueBeam and two Clinac machines. Using the same phantom, digital WL tests with 16 combinations of gantry and collimator angles were performed to locate the radiation isocenter. The offsets between the IsoCal‐calibrated image centers and the WL radiation isocenter were found to be within 0.4 mm on the four linacs in this study. When IsoCal was turned off, the maximal offsets of the image centers were greater than 1.0 mm on the two Clinac machines. The method developed in this study can be used as a vendor‐independent quality assurance tool to assess the isocentricity of the image centers and radiation central axes.

## 1 INTRODUCTION

Spatial accuracy has always been a critical aspect of quality assurance (QA) in the field of radiation therapy (RT). To ensure spatial accuracy, image guidance (IG) is often utilized to align radiation and the patient. IG has advanced rapidly in recent years: from megavoltage (MV) to kilovoltage (kV) x rays, from 2D to 3D and 4D imaging, and from radiography to magnetic resonance imaging. MV/kV planar imaging and cone beam computed radiography (CBCT) systems have become widely available on modern linear accelerators (Linacs). While the IG systems may vary in physical mechanism, they must provide accurate spatial presentations of the patient in the reference frame of treatment machines. To this end, a key requirement for the IG systems is that the image coordinates should match the treatment coordinates.^1^, ^2^ Specifically, the origin of the image coordinates, referred to here as the image center, should coincide with the isocenter of the treatment machines, or the radiation isocenter.

On Varian and Elekta linacs, the coincidence between the image centers and the radiation isocenter is often compromised by various imperfections in the IG systems and the linac itself. These imperfections include but are not limited to mechanical sag of image source and image detector, imperfect rotation of gantry, miscalibration of collimator, and misalignment of room lasers.^2^, ^3^, ^4^, ^5^, ^6^ As a result, the image centers deviate from the radiation isocenter. The amount of deviation is often complicated and dependent on the gantry rotation. In addition, the image centers can drift over time. To ensure IGRT accuracy, linac manufacturers have developed different approaches to compensate for the image center deviations. On Elekta linacs, the IG systems can be geometrically calibrated by imaging a ball‐bearing (BB) phantom positioned at or near the linac radiation isocenter and analyzing the images with the x ray volume imaging (XVI) software.^7^, ^8^ On Varian Clinac‐series linacs, the accuracies of image centers are affected by several geometric calibrations including mechanical calibration of imaging arms, central axis alignment of image detectors, CBCT geometric calibration, and more recently IsoCal calibration.^9^ Among these calibrations, only IsoCal relates the image centers directly to the radiation isocenter, that is, not through the room lasers or the crosshair of light fields. IsoCal is designed to be carried out as the final step of all geometric calibrations of the IG systems. The goal of IsoCal calibration is to refine the image centers to match the linac radiation isocenter. IsoCal is made optional on Varian Clinac‐series and mandatory on the newer linac models, that is, the TrueBeams. While conceptually identical, IsoCal calibrations are implemented differently on Clinac and TrueBeam linacs. The IsoCal procedure starts with acquiring 2D MV, 2D kV, and 3D CBCT images of a cylindrical phantom containing multiple BBs and a collimator insert.^10^, ^11^ The sampling interval for the gantry rotation is 45° on the Clinacs or 3° on the TrueBeams. The acquired images are then analyzed in the IsoCal software to derive corrections to the image centers that are specific to the gantry angles and the imaging modalities. The corrections are applied later in either of the following two ways. On Clinacs, the correction is written to the DICOM header of the later acquired images. When the image is displayed, the correction is applied to the digital graticule via software. On TrueBeams, the correction is applied physically to the robotic arm before the image is taken, thus requiring no software shift to the digital graticule. The primary focus of this study is to evaluate the image center accuracies after the IsoCal calibration.

Despite the different approaches taken by linac manufacturers, the effectiveness of these approaches has not been thoroughly investigated by independent studies. A primary technical barrier is the lack of precision tools needed in evaluating the small, often submillimeter, deviations of the image centers from the radiation isocenter. The traditional IGRT QA method uses a cube phantom that is positioned at the mechanical isocenter using the room lasers.^5^ The accuracy of this method is inherently limited due to the uncertainty in the room lasers. The recent Machine Performance Check (MPC) method is reported to have high accuracy;^12^, ^13^ however, it is developed by the same linac vendor (Varian) and thus cannot be regarded as an independent QA method. In this study, we independently assess the effectiveness of the Varian IsoCal technique. We employ the digital Winston‐Lutz (WL) test method, which has been demonstrated to have submillimeter accuracy.^4^, ^6^, ^14^, ^15^, ^16^ This method measures the image center accuracies directly against the radiation isocenter. Unlike the traditional WL test, the digital WL test does not require a precision linear stage to adjust the phantom position iteratively to the radiation isocenter. Therefore, the digital WL test is simple and fast in terms of its phantom and the setup. A previous study used the digital WL test to verify the IsoCal effectiveness on Varian Clinac machines.^10^ The study showed that IsoCal increased the coincidence between the 2D image centers and the radiation isocenter to within 0.6 mm. The digital WL in that study used four gantry angles and a single collimator angle, that is, the collimator rotation was not considered. In this study, we re‐design the digital WL test by employing more gantry and collimator angles to achieve higher accuracy in localizing the radiation isocenter. We implement this method to evaluate the IsoCal effectiveness on Varian TrueBeam machines. Furthermore, we include the evaluation of CBCT image centers on both Clinac and TrueBeam machines in this study.

## 2 MATERIALS AND METHODS

### 2.A IsoCal on Varian clinac machines

The theory and procedure of IsoCal for Varian Clinac machines have been previously described.^10^, ^11^ First, a cylindrical phantom containing 16 BBs was positioned at the mechanical isocenter using the room lasers. An aluminum plate with a steel pin was inserted in the gantry accessory slot. Four MV images of the phantom were obtained at collimator angles (195°, 270°, 0°, 90°; Varian IEC 601‐2‐1 scale) and a fixed gantry angle (0°) to establish the central axis (CAX) of the radiation beam at the given gantry angle. Subsequently, 8 MV images of the phantom were acquired at various gantry angles (225°, 270°, 315°, 0°, 45°, 90°, 135°, 180°) and a fixed collimator angle (90°) to establish the radiation isocenter. Finally, 8 kV images and a CBCT scan of the phantom were obtained. Using these images and the Varian IsoCal software, the 2D MV, 2D kV, and CBCT images centers were localized relative to the radiation isocenter. The offsets of the image centers were used to create a system file in XML format. In subsequent imaging (MV, kV, or CBCT), the XML file was used to register an image center correction to the image's DICOM header. The correction was applied to the digital graticule when the image was displayed in the Varian OBI console, or in a third party software such as MOSAIQ (Elekta AB, Stockholm, Sweden).

### 2.B IsoCal on Varian TrueBeam machines

The hardware of the IsoCal system on TrueBeam linacs is identical to that on Clinac linacs. Two automatic procedures, IsoCal calibration and IsoCal verification, are associated with the IsoCal system. In both procedures, we acquired 4 MV images of the phantom at different collimator angles (195°, 270°, 0°, 90°) and a fixed gantry angle (180°). We then acquired a series of MV images and kV images of the IsoCal phantom with a full gantry rotation, approximately one image every 3° gantry angle. Similar to the IsoCal system on Clinac, the IsoCal calibration was to determine the corrections required to align the centers of the kV and MV images to the radiation isocenter. At the end of IsoCal calibration, the correction data were stored in a configuration file in the Varian on‐board imaging system. After the IsoCal calibration was completed, the IsoCal corrections were subsequently applied by correcting the lateral and longitudinal MV or kV imager positions as a function of gantry angles before the images were taken, and thus no shifts of the digital graticules were needed. This was in contrast to procedures used with the IsoCal on Clinac where imagers were not shifted but the corrections were applied by shifting the digital graticules of the acquired images.

On TrueBeam linacs, the IsoCal verification procedure was performed to validate the IsoCal calibration. The IsoCal verification procedure was the same as the IsoCal calibration procedure except that the IsoCal corrections were applied to MV and kV imagers. The residual corrections from the IsoCal verification should be close to zero.

### 2.C Digital Winston‐Lutz test

**Figure 1:**
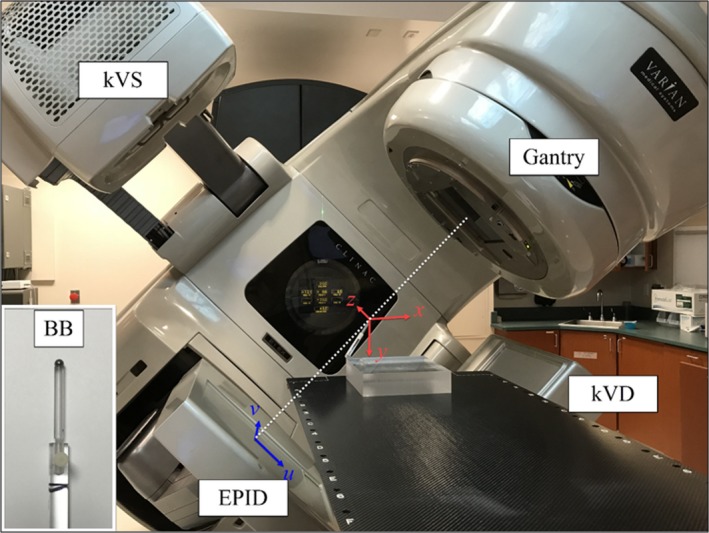
Phantom setup and the image coordinate systems. A ball‐bearing (BB) is placed approximately at the linac isocenter. The center of the BB serves as the origin of the stationary x‐y‐z coordinate system and the origin of the rotational u‐v coordinate system.

The WL phantom included a tungsten sphere of 6.5 mm in diameter glued on an acrylic rod, which was screwed into an acrylic block (Fig. 1). The phantom was placed on the treatment table and kept stationary during the entire image acquisition. The BB was placed near the linac isocenter (within ±3 mm in each direction) using the guidance of room lasers. The center of the BB was used a reference point to which the radiation isocenter and the image centers were localized. Thus, there was no need to place the BB exactly at the radiation isocenter. Two coordinate systems were used in this study. The first coordinate system x‐y‐z was static with the origin defined at the BB center. The second coordinate system u‐v was defined for the 2D MV and 2D kV images (Fig. 1). The u‐v coordinate system rotated with the MV source or the kV source. The origin of u‐v coordinates was defined at the projection of the BB center on the imager. The values of u‐v coordinates were scaled to the isocenter plane.

To locate the radiation isocenter using the WL method, the BB phantom was imaged with a 10 × 10 cm^2^ square MV field shaped by the multi‐leaf collimator (MLC). The images were acquired at eight gantry angles (225°, 270°, 315°, 0°, 45°, 90°, 135°, 180°) and two opposing collimator angles (90° and 270°). A total of 16 MV images were obtained to compute the location of the radiation isocenter. In previous studies, only 4 MV images (four cardinal gantry angles and one collimator angle 0°) were employed for simplicity.^6^, ^10^ The use of opposing collimator angles in this study was intended to improve the accuracy of radiation isocenter localization.

The MV images were processed with an in‐house MATLAB (MathWorks Inc., Natick, MA) program. The details of the algorithm were reported previously.^16^ Briefly, the radiation field center in each MV image was located relative to the BB center. Then the 16 radiation CAX were reconstructed in the 3D x‐y‐z space. Finally the radiation isocenter was determined as the point that had the minimal average distance from all CAX. With eight gantry angles and two opposing collimator angles, the uncertainty in the resulting radiation isocenter was estimated to be less than 0.2 mm.^16^ For each gantry angle and collimator angle, we also computed the distance of the radiation CAX to the WL radiation isocenter. The maximal and mean distances for the 16 radiation CAX were used to characterize the size of the isocenter sphere. The gantry sag was computed as the longitudinal (v or z direction) shifts of the CAX during the gantry rotation. The couch rotation is not considered in this study because (a) the IsoCal procedure does not include the couch rotation, (b) the mechanical walkout during couch rotation is patient‐dependent, that is, varying with the patient weight and how the weight is distributed on the couch, (c) the couch walkout can be corrected by realigning the couch with the calibrated ceiling lasers.

The digital WL tests were performed twice on each of the two Varian TrueBeams and two Varian Clinac 21EX linacs.

### 2.D IsoCal corrections to image centers

**Figure 2:**
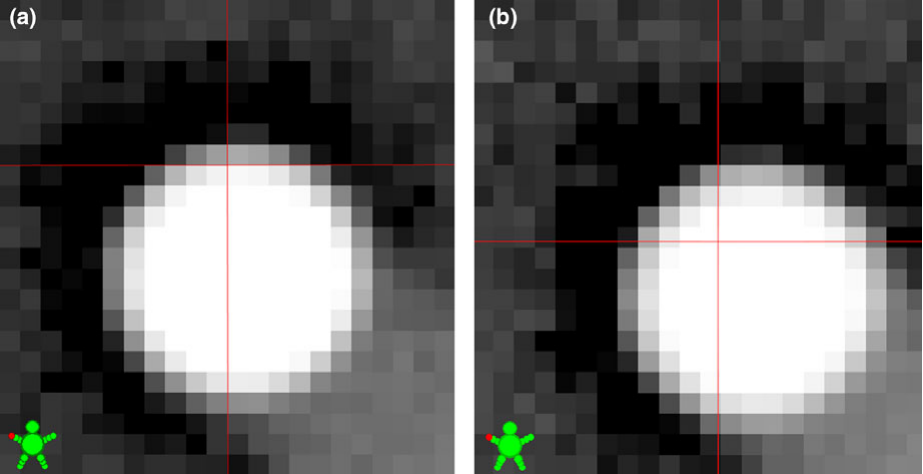
Close‐up view of the image center or digital graticule (red cross) relative to the pixelated MV projections of the WL phantom. IsoCal correction was disabled in (a) or enabled in (b). The MV images were acquired on a Varian Clinac 21EX machine.

Following the acquisition of the 16 MV images, four kV images (source angles 270°, 0°, 90°, 180°) and one CBCT scan of the BB phantom were acquired. If the IsoCal correction was disabled, the image centers in each MV image, kV image, or the CBCT images were defaulted to the center of image matrix (no manual offsets of EPID and kVD were used throughout this study). For example, there were 1024 pixels in the u‐dimension of MV images. Assuming that each pixel was a small rectangle with no gap or overlap between its neighbors, the default image center position was located exactly between the 512th pixel and the 513th pixel [Fig. 2(a)]. The default image center position in the other dimensions was similarly defined. On Varian TrueBeam linacs, the image centers or digital graticules were defined at the default image centers.

On Varian Clinacs, if the IsoCal was enabled, the image centers needed to be corrected by an amount that was derived from the IsoCal calibration [Fig. 2(b)]. The amount of correction was found in the DICOM header of the acquired images. For 2D MV or kV images, the DICOM tag “X‐Ray Image Receptor Translation” (IRT) defined the detector panel position. The IsoCal correction (∆_IsoCal_) was

(1) $$\Delta_{\mathrm{IsoCal}}=-\frac{\mathrm{IRT}}{SID/SAD},$$

where SID was the source‐to‐imager distance, SAD was the source‐to‐axis distance, and the ratio SID/SAD was a scaling factor. For CBCT images, the IsoCal correction was derived in three steps from the header of the DICOM RT Structure file that accompanied the CBCT image files. First, the Acquisition Isocenter (AcqIso) position was found in the tag “ROI Contour Sequence/Item 1/Contour Sequence/Item 1/Contour Data”. Second, the Acquisition Isocenter coordinates were transformed from the patient‐based coordinate system to the equipment‐based coordinate system, using a rotation matrix:

(2) $$\left(\begin{array}{c} AcqIso_{x}^{\prime }\\ AcqIso_{y}^{\prime }\\ AcqIso_{z}^{\prime } \end{array}\right)=\left[\begin{array}{ccc} \cos(\theta_{t}) & 0 & -\sin(\theta_{t})\\ 0 & 1 & 0\\ \sin(\theta_{t}) & 0 & \cos(\theta_{t}) \end{array}\right]\left(\begin{array}{c} AcqIso_{x}\\ AcqIso_{y}\\ AcqIso_{z} \end{array}\right),$$

where *θ*_t_ was the couch angle in degrees when the CBCT scan was acquired. If *θ*_t_ = 0°, AcqIso′ = AcqIso. Third, the IsoCal correction was computed as

(3) $$\left(\begin{array}{c} \Delta_{IsoCal,x}\\ \Delta_{IsoCal,y}\\ \Delta_{IsoCal,z} \end{array}\right)=\left(\begin{array}{c} AcqIso_{x}^{\prime }\\ AcqIso_{y}^{\prime }\\ AcqIso_{z}^{\prime } \end{array}\right)+\left(\begin{array}{c} FOV_{x}/2\\ FOV_{y}/2\\ ST/2 \end{array}\right),$$

where FOV was the field‐of‐view, and ST was the slice thickness of the CBCT images.

The final IsoCal‐corrected image center position was the sum of the default image center position and the IsoCal correction described above. After the BBs were detected in the MV, kV, or CBCT images, the image centers were localized relative to the BB center. Given the WL radiation isocenter position as determined from Section 2.C above, the image center offsets relative to the WL radiation isocenter could be easily computed.

### 2.E Varian linacs evaluated

We selected two Varian TrueBeam and two Varian Clinac 21EX machines for the IsoCal evaluation. Durations of operation were approximately 11 yr for Clinac1 and Clinac2, and 4.5 and 2.0 yr for TrueBeam1 and TrueBeam2, respectively. The IsoCal procedure was performed on each linac according to the methods described in Sections 2.A and 2.B above. Then the digital WL test and the imaging (2D kV, CBCT) of the phantom were performed according to the methods described in Sections 2.C and 2.D. To investigate the reproducibility of the IsoCal effects, we repeated the measurements after an interval of approximately 1 month.

## 3 RESULTS

**Figure 3:**
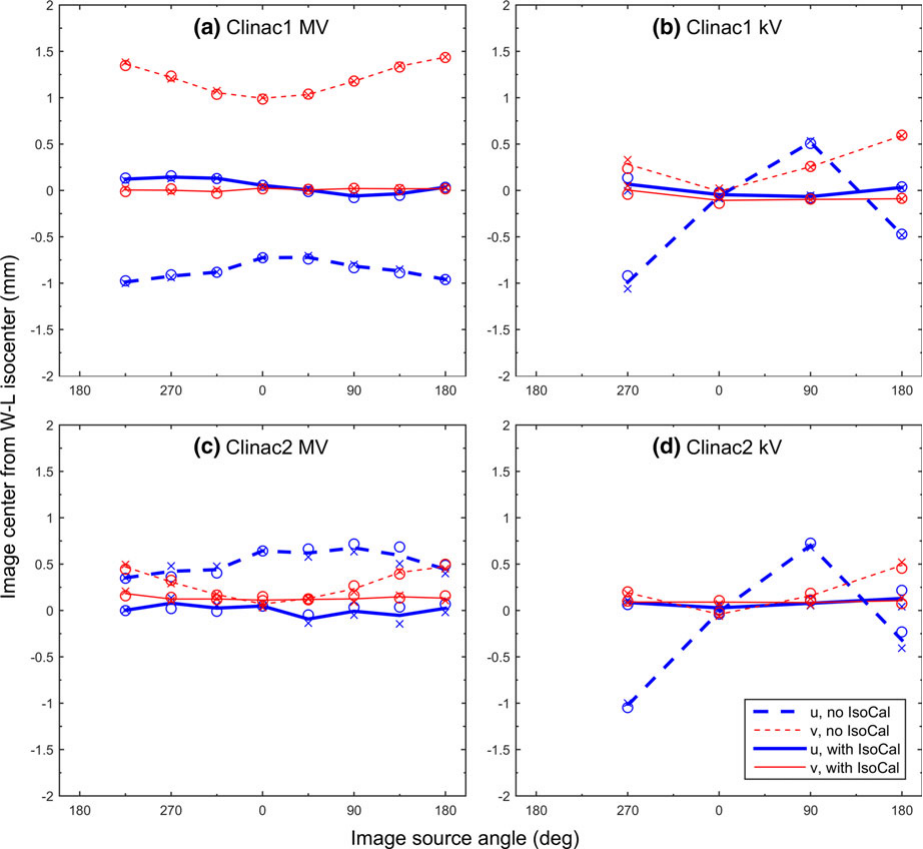
Offsets of 2D MV (a and c) and 2D kV (b and d) image centers from the WL isocenter on the two Clinac machines. U and v were the lateral and longitudinal dimensions, respectively, on the image plane. IsoCal was turned on (solid lines) or off (dashed lines). The measurements were made twice (circles and crosses).

Figure 3 shows the image center offsets from the WL isocenter measured on Clinac1 and Clinac2. Without IsoCal, the largest lateral and longitudinal offsets were 1.0 mm (u) and 1.4 mm (v) for the MV images, and 1.1 mm (u) and 0.6 mm (v) for the kV images, for Clinac1 [Figs. 3(a) and 3(b)]. The offsets were reduced to within 0.2 mm for both MV and kV images when the IsoCal corrections were applied. Similar reductions in image center offsets were also seen on Clinac2 [Figs. 3(c) and 3(d)]. The IsoCal‐corrected image centers were within 0.2 mm from the WL isocenter. The differences between the repeat measurements were on the order of 0.1 mm, indicating the high stability of linac mechanical movements over time and high precision of the digital WL tests.

**Figure 4:**
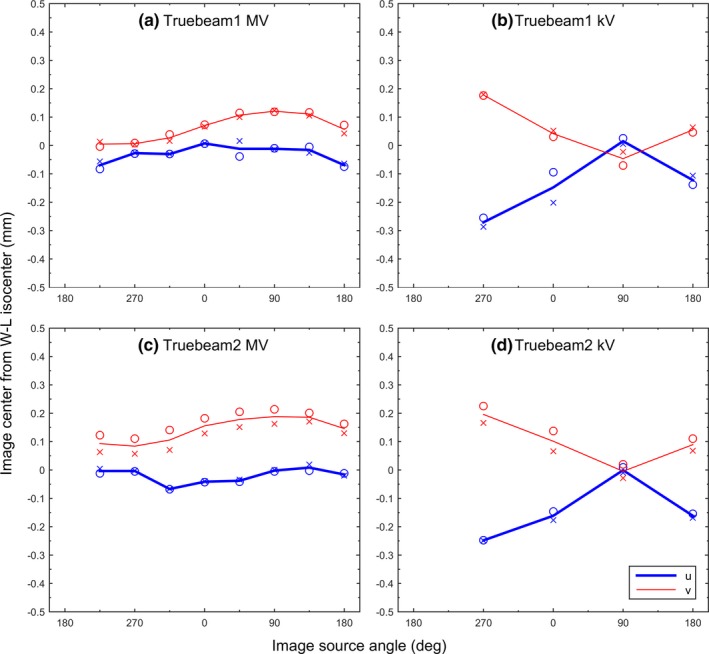
Offsets of 2D MV (a and c) and 2D kV (b and d) image centers from the WL isocenter on the two TrueBeam machines. The measurements were made twice (circles and crosses).

Figure 4 shows the measured image center offsets for the two TrueBeam machines. Since IsoCal calibration is required on TrueBeam machines, only the IsoCal‐corrected image centers were studied. The largest lateral and longitudinal offsets were 0.1 mm (u) and 0.2 mm (v) for the MV images, and 0.3 mm (u) and 0.2 mm (v) for the kV images for both TrueBeam1 [Figs. 4(a) and 4(b)] and TrueBeam2 [Figs. 4(c) and 4(d)]. The data from the repeat measurements were highly reproducible (<0.1 mm variations).

**Figure 5:**
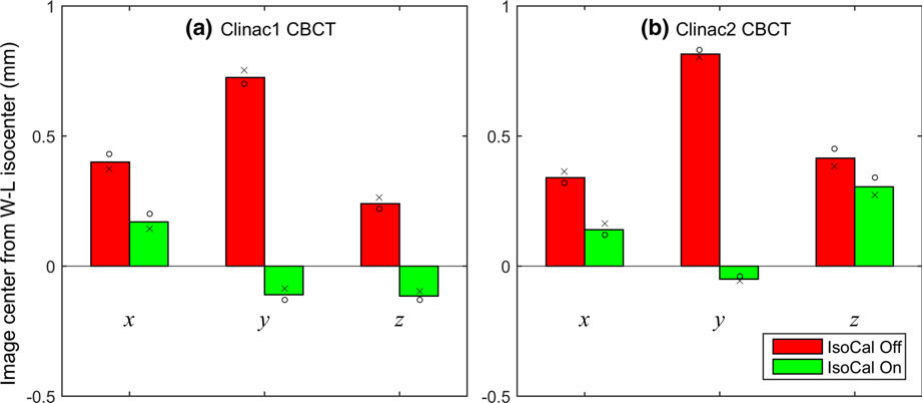
Offsets of CBCT image center from the WL isocenter on Clinac1 (a) and Clinac2 (b). x, y, z were the lateral, vertical, and longitudinal dimensions of the room coordinate system. IsoCal was turned on (green) or off (red). The measurements were made twice (circles and crosses).

**Figure 6:**
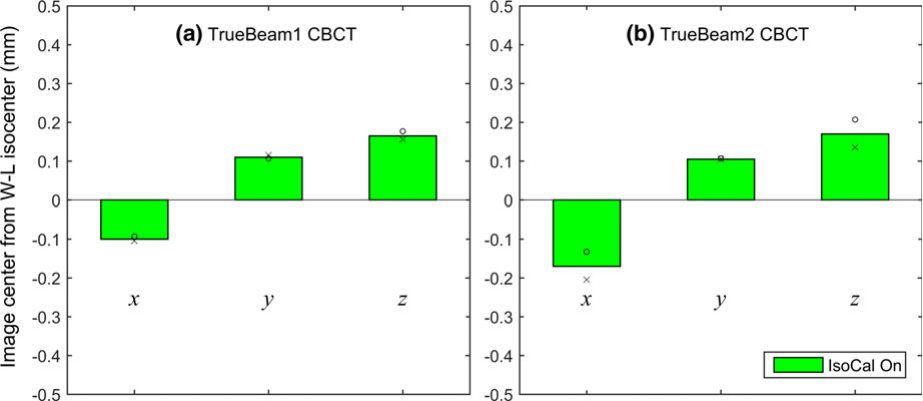
Offsets of CBCT image center from the WL isocenter on TrueBeam1 (a) and TrueBeam2 (b). The measurements were made twice (circles and crosses).

The IsoCal effects on CBCT image centers are illustrated in Figs. 5 and 6. Without IsoCal, the largest offsets were 0.7 and 0.8 mm in the vertical (y) direction for Clinac1 [Fig. 5(a)] and Clinac2 [Fig. 5(b)], respectively. With IsoCal, the largest offsets in CBCT image centers were decreased to 0.2 mm for Clinac1 and 0.3 mm for Clinac2. On both TrueBeam machines, the largest offsets in CBCT image centers were 0.2 mm, with the IsoCal corrections applied inherently (Fig. 6).

**Figure 7:**
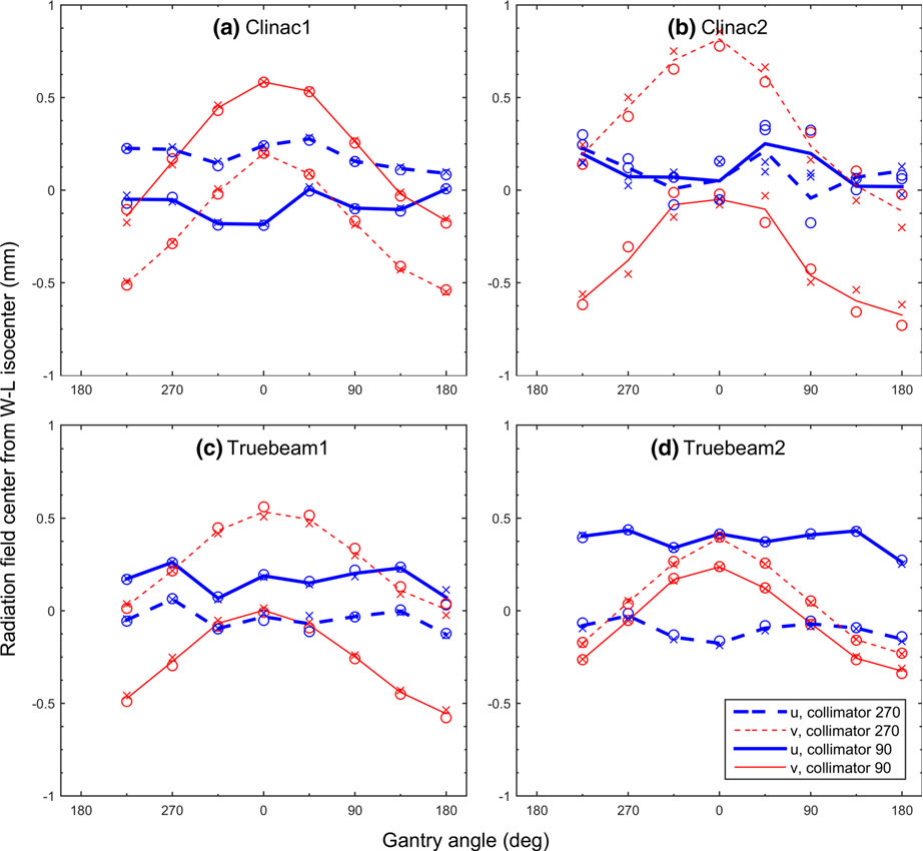
Offsets of radiation CAX from the WL radiation isocenter on Clinac1 (a), Clinac2 (b), TrueBeam1 (c), and TrueBeam2 (d). The measurements were made twice (circles and crosses).

A useful by‐product of doing the WL test is to quantitate the wobble of radiation fields. Figure 7 shows the wobble of radiation fields around the WL isocenter as a function of gantry angle and collimator angle. The maximal distances of the CAX wobble, or the radii of isocenter sphere, were 0.58 and 0.83 mm for Clinac1 [Fig. 7(a)] and Clinac2 [Fig. 7(b)], respectively (Table 1). The radii of the isocenter sphere were 0.57 and 0.50 mm for TrueBeam1 [Fig. 7(c)] and TrueBeam2 [Fig. 7(d)], respectively. On each linac, the wobble in the lateral (u) dimension was approximately constant in comparison to the sinusoidal patterns for the wobble in the longitudinal (v) dimension. The gantry sag, or the longitudinal movement of CAX, commonly existed on both types of machines. The ranges of gantry sag were 0.56 and 0.59 mm on the TrueBeam machines, which were smaller than those on the Clinac machines, 0.75 and 0.79 mm. The gantry sag measured on the Clinac machines was consistent with the values previously reported in the literature.^2^, ^4^, ^17^

**Table 1:** Measured distances of the radiation CAX from the WL isocenter and the ranges of gantry sag. [mean or maximum (mm) ± 1 standard deviation (SD)]

	Distance = |CAX‐WL isocenter|		Gantry Sag
	Mean	Max	Range
Clinac1	0.35 ± 0.16	0.58 ± 0.01	0.75 ± 0.01
Clinac2	0.43 ± 0.26	0.83 ± 0.04	0.79 ± 0.19
TrueBeam1	0.31 ± 0.18	0.57 ± 0.02	0.56 ± 0.01
TrueBeam2	0.33 ± 0.15	0.50 ± 0.01	0.59 ± 0.04

## 4 DISCUSSION

In this study, we evaluated the IsoCal effectiveness on Varian linacs using an independent digital WL test method. Clearly, IsoCal was effective in bringing the coincidences between the image centers and the radiation isocenter down to 0.4 mm or less on all four linacs we studied. This level of image center accuracy is well within the AAPM recommended tolerance of 1 mm for stereotactic types of machines.^1^ If the IsoCal corrections were not applied, the image center inaccuracies could exceed 1 mm for the two Varian Clinac 21EX linacs (Fig. 2). Thus, IsoCal has the capability to significantly improve IGRT accuracy and potentially enable the linacs for stereotactic types of radiation therapy.

The IsoCal calibration consists of vendor‐provided hardware, software, and methodology. Many technical details and much of the interpretation of the IsoCal results appear as a “black box” to clinical medical physicists. Thus, it is imperative to have an independent, more transparent method to assess the effectiveness of IsoCal. The WL test has been established for decades as a precise way of localizing the radiation isocenter of a linac. The concepts of using a simple BB phantom and circular or square radiation fields in the WL test are well understood. In this study, we used the WL test to locate the radiation isocenter, which was independent of the IsoCal methodology. Unlike in some previous studies,^12^ we did not use Varian toolkits (eg, IsoLock procedure, MarkerBlock phantom) for the WL test. Nonetheless, the close agreements between the WL isocenter and the IsoCal‐corrected image centers indicated that the WL isocenter matched well with the isocenter that was derived from the IsoCal procedure. With the known WL isocenter location, we were able to extend our method into other clinical applications. For example, we evaluated the image center accuracy of an ExacTrac IGRT system (Brainlab AG, Munich, Germany), which was installed on one of the linacs (data not shown). This would be not readily achievable using IsoCal alone.

The coincidence between the IsoCal‐corrected image centers and the WL isocenter was within 0.4 mm on the Varian Clinac and TrueBeam machines in this study. This level of coincidence was better than the 0.6 mm range reported in a previous study,^10^ which was based on a simplified WL test and Varian Clinac machines only. The simplified WL test involved MV imaging at four cardinal gantry angles and one collimator angle of 0°. In this study, we used MV imaging at eight gantry angles and two opposing collimator angles of 90° and 270°. The increased number of CAX samples reduces the random errors in locating the WL isocenter.^16^ The use of opposing collimator angles also reduces the systematic errors in determining the longitudinal (z) position of the WL isocenter. In the vertical (y) direction, the WL isocenter determined in this study was systematically higher than the WL isocenter if the simplified WL test (collimator = 0°; gantry = 0°, 90°, 180°, 270°) was used. Our measurements on Clinac1 and Clinac2 showed that the WL isocenter would be lower by 0.19 and 0.21 mm, respectively, if the simplified WL tests were used. Similarly, on TrueBeam1 and TrueBeam2, the WL isocenter would be lower by 0.20 and 0.24 mm if the simplified WL tests were used. The lower WL isocenters with collimator 0° were presumably due to the gravitational pull on the MLC leaves when the gantry was at 90° and 270°, or other oblique angles. This effect had also been observed in a previous study.^16^ Therefore, the range of the WL isocenter variations with various collimator angles was approximately 0.2 mm for the Clinac and TrueBeam linacs in this study. We estimated that the WL isocenter determined with collimator 90° and 270° would be roughly 0.1 mm higher than the “true” radiation isocenter if the collimator 0° data were included in the analysis.

In IsoCal procedures, the MLC leaf positions were not used in isocenter localization. Thus, the gravitational pull on the MLC leaves had no effect on the IsoCal isocenter. This might partially explain the better image center vs WL isocenter coincidence in this study (collimator = 90° and 270°, no gravitational pull on the MLC leaves) compared to the previous study (collimator = 0°, MLC leaves subject to gravitational pull).^10^ Furthermore, we estimated that the IsoCal corrected image centers were within 0.5 mm of the “true” radiation isocenter. This estimation was based on ≤0.4 mm coincidence between the WL isocenter and the image centers, 0.1 mm systematic errors in the WL isocenter in this study, and 0.1 mm random errors.

Finally, the ways that Varian implements the IsoCal corrections are worthy of discussion. On the Clinac series, the IsoCal correction is imbedded in the DICOM header of the acquired images. Interpretation of this information is not a straightforward task for the end users, even with the guidance of the vendor's DICOM Conformance Statement or system manuals. On the TrueBeam machines, the IsoCal correction is applied to adjust the imager position. Once the image is taken, no extra shift is needed to correct the image center. This latter practice eliminates the need of interpretation or guesses by the third party and thus is more desirable in terms of preventing potential errors in patient positioning.

## 5 CONCLUSIONS

We investigated the effectiveness of IsoCal on Varian's Clinac and TrueBeam machines. The 2D MV, 2D kV, and CBCT image centers were found to coincide with the WL radiation isocenter within 0.4 mm on both types of Varian machines. If not corrected by IsoCal, the image centers on the Clinac machines could be more than 1 mm off from the WL isocenter. Our independent study indicates that IsoCal is essential in improving the image center accuracy on the Varian linacs to within 0.5 mm accuracy. The method developed in this study can be used as a vendor‐independent QA tool to assess the isocentricity of the image centers and radiation central axes.

## CONFLICT OF INTEREST

The authors do not have any conflicts of interest to declare. This work was not sponsored by Varian Medical Systems.
